# Effect of chemoprevention by low-dose aspirin of new or recurrent colorectal adenomas in patients with Lynch syndrome (AAS-Lynch): study protocol for a multicenter, double-blind, placebo-controlled randomized controlled trial

**DOI:** 10.1186/s13063-020-04674-8

**Published:** 2020-09-04

**Authors:** Adil Soualy, David Deutsch, Mourad Benallaoua, Amal Ait-Omar, Florence Mary, Sabine Helfen, Marouane Boubaya, Vincent Levy, Robert Benamouzig, the AAS-Lynch group, Robert Benamouzig, Florence Mary, Amal Aït Omar, Mourad Benallaoua, Sabine Helfen, Semaher Al-Khafaji, Noémie Demare, Géraldine Perkins, Pierre Laurent-Puig, Jean-Christophe Saurin, Naouele Raby, Laurence Venat-Bouvet, Corinne Penaud, Delphine Bonnet, Virginie Sicart, Chloé Pomes, Thierry Lecomte, Claire Jollivet, Morgane Caulet, Stanislas Chaussade, Marion Dhooge, Fanny Maksimovic, Philippe Grandval, Sylvie Olschwang, Maud Saussereau, Jérôme Bellanger, Anne Netter-Coti, Hélène Delhomelle, Bruno Buecher, Lydia Mehdi, Sophie Lejeune, Afane Brahimi, Stéphane Cattan, Laurence Bellengier, David Tougeron, Sandrine Rafert, Emmanuelle Barouk Simonet, François Cornelis, Anna Serova-Erard, David Malka, Paul Gesta, Jeanne Oddoz, Véronique Mari, Samuel Lesourd, Gaëlle Kergoat, Louise Crivelli, Iradj Sobhani, Aurélien Amiot, Côme Lepage, Laurence Faivre-Olivier, Jean-Louis Jouve, Antoine Drouillard, Nora Perot, Marc Bardou, Sophie Nambot, Nadia Mekahli, Alain Lortholary, Carole Lenne, Jean-Paul Lagasse, Brahim Ouahrani, Thierry Frebourg, Nathalie Parodi, Maud Branchaud, Françoise Desseigne, Elodie Grinand, Olivier Ingster, Benoit Semelin, Francine Fein, Nelson Lourenco, Thomas Aparicio, Mathilde Brasseur, Anthony Lopez

**Affiliations:** 1 Service de Gastroentérologie, Hôpital Avicenne, Université Paris 13, 125 Rue de Stalingrad, 93000 Bobigny, France; 2 Unité de Recherche Clinique, Hôpital Avicenne, Bobigny, France

## Abstract

**Abstract:**

Lynch syndrome (LS) is the most common cause of inherited colorectal cancer (CRC) and confers a high lifetime risk of CRC estimated to be up to 60%. Colonoscopy is recommended every 2 years in LS patients above the 20–25-year-old age bracket, and every year when colonic neoplasia has been detected. Efficient chemoprevention has the potential to represent a cost-effective intervention in these high-risk patients and could allow a delay in colonoscopy surveillance. Several epidemiological studies have shown that regular use of low dose aspirin is associated with a 20 to 30% reduction in the risk of sporadic colonic adenomas and colorectal cancer regardless of family risk. However, in recent large randomized trials in specific populations, aspirin use showed no protection for colorectal cancer. A prospective randomized CAPP-2 trial evaluated the effect of aspirin use in LS patients. The primary analysis of this trial showed no significant decrease in CRC in LS patients under daily aspirin. However, a preplanned secondary analysis after an extended follow-up showed a significant reduced risk of CRC in the aspirin group in the per-protocol analysis. The real effect and clinical benefit of aspirin are still to be consolidated in this population. The AAS-Lynch trial—a prospective, multicentric, double-blind, placebo-controlled, randomized clinical trial—was designed to investigate if daily aspirin therapy, at a dose of 100 or 300 mg, would decrease the occurrence or recurrence of colorectal adenomas in patients under 75 years of age, compared with placebo.

**Trial registration:**

ClinicalTrials.gov NCT02813824. Registered on 27 June 2016. The trial was prospectively registered.

## Introduction

Lynch syndrome is the most common cause of inherited colorectal cancer. It is linked to germline alterations in mismatch repair genes that confer a high lifetime risk of colorectal cancer estimated to be as high as 60%. Most colorectal cancers arise from asymptomatic adenomas. Early detection and removal of adenomas can prevent their transformation into cancers. Colonoscopy is recommended every 2 years in Lynch syndrome patients above the 20–25-year-old age bracket, and every year when colonic neoplasia has been detected [1]. Efficient chemoprevention has the potential to represent a cost-effective intervention in these high-risk patients and could allow a delay in colonoscopy surveillance. Aspirin therapy decreases the risk of colorectal cancer in experimental models. Multiple mechanisms have been reported to explain this effect [2]. Several epidemiological studies have shown that the regular use of low dose aspirin (75 to 300 mg/d) is associated with a 20 to 30% reduction in the risk of sporadic colonic adenomas and colorectal cancer regardless of family risk [3]. Seven randomized controlled trials (RCT) evaluating adenoma recurrence are available [4–10]. Five out of seven trials showed a significant decrease in neoplasia recurrence [4–6, 8, 10]. A meta-analysis of four of these studies confirmed a decrease in colorectal adenoma recurrence [11]. In a pooled analysis of cardio-vascular prevention RCTs, and in a meta-analysis, daily aspirin was associated with a reduced risk of CRC and CRC associated mortality [12]. However, in two large, recent, randomized trials in diabetic patients and in healthy elderly patients, aspirin use showed no protection for colorectal cancer [13, 14]. The Colorectal Adenoma/carcinoma Prevention Programme 2 (CAPP-2) evaluated aspirin 600-mg (300 mg b.i.d) effect in LS patients. The primary analysis of this prospective randomized trial showed no significant decrease in CCR risk in LS patients under daily aspirin [15]. However, a preplanned secondary analysis after an extended follow-up (mean 56 months) showed a significantly reduced risk of CRC in the aspirin group in the per-protocol analysis [16]. In this study, the endoscopic follow-up was sub-optimal with a relatively low detection rate of colorectal neoplasia compared to the actual reported rate [17], or the chromo-endoscopy derived rate [18]. The exact effects and clinical benefits of aspirin therapy are still to be consolidated in LS patients. Notably, aspirin’s preventive benefits are expected to outweigh its putative adverse effects in these high-risk patients.

We designed the AAS-Lynch trial, which is a prospective, multicentric, double-blind, placebo-controlled, randomized clinical trial, to investigate whether the daily use of aspirin, at a dose of 100 or 300 mg, in LS patients under 75 years of age, would decrease the occurrence or recurrence of colorectal adenomas, compared with placebo. This trial will also investigate the role of the gut microbiota in adenoma apparition or recurrence in LS patients.

## Methods

### Eligibility criteria

Eligible subjects are adults aged 18 to 75 years with LS carrying a defined “mismatch repair” gene mutation or, when no characteristic alteration had been found, with a personal or family history of LS according to modified Amsterdam criteria (CRC diagnosed before 50 years of age; presence of synchronous or metachronous colorectal or other LS-related tumors regardless of age; CRC with MSI-high histology diagnosed in a patient who is younger than 60 years of age; CRC diagnosed in a patient with one or more first-degree relatives with a LS-related cancer which was diagnosed under 50 years of age; and CRC diagnosed in a patient with two or more first- or second-degree relatives with LS-related cancer regardless of age). Detailed inclusion and exclusion criteria are presented in Table 1.

**Table 1:** AAS-Lynch eligibility criteria

Inclusion criteria	Exclusion criteria
- Men and women with Lynch syndrome with an alteration of “mismatch repair” genes or when no characteristic alteration has been found, with a personal or family history of Lynch syndrome according to modified Amsterdam criteria. - Aged over 25 or 18 years of age in cases with an early family history, with an indication for colonoscopic surveillance every 2 years - Under 75 years old. - Performing a colonoscopy within 180 days before inclusion, with removal of all endoscopically resectable polyps. - No regularly use of aspirin throughout the (7 consecutive days for at least 3 weeks per year or more than 21 days throughout the year). - Effective contraception for women of, defined by a hormonal method or IUD or surgical sterilization of the patient or her partner - Patient affiliated to a social security (excluding AME) or entitled - Patient who has given his consent to participate by signing the consent of the study	- Total colectomy - Adenomatous polyposis associated with known alteration of APC gene or MYH gene - Allergy to aspirin (including a history of asthma induced by the administration of salicylates or substances of similar activity, especially nonsteroidal anti-inflammatory drugs) - Allergy known to indigo carmine used for chromoendoscopy - Indication of prolonged treatment (prevention of atheromatous risk) or repeated treatment (recurrent migraine) with aspirin or other nonsteroidal anti-inflammatory drug (NSAID) - Abnormality of hemostasis or coagulation (including gastrointestinal hemorrhage, history of hemorrhagic stroke and thrombocytopenia) - Indication of long-term treatment with anticoagulant antiplatelet agents, anagrelide or uricosuric agents- History of digestive ulcer - Digestive hemorrhage related to ulcerative disease in the 12 months prior to inclusion - Gastric pathology deemed significant by the investigator and not corrected by appropriate treatment - Uncontrolled high blood pressure - Renal insufficiency (creatinine clearance < 30 ml/min) - Severe hepatic insufficiency (defined by a PT < 70%) - Severe uncontrolled heart failure - Known failure of G6PD deficit - Recent diagnosis of colorectal cancer requiring specific management - Presence of menorrhagia and not corrected by appropriate treatment - Pregnancy or breastfeeding - Any disease that may interfere with the follow-up provided by the protocol or invalidate the proper understanding of the protocol information and informed consent - Patient under the protection of justice - Participation in another therapeutic clinical trial in the 12 weeks prior to inclusion

### Intervention

A centralized randomization with stratification by age (< or ≥ 45 years) and center will be performed. It was not possible to obtain the 2 doses of aspirin (100 and 300 mg) with the same aspect; therefore, 2 different types of placebo tablets were used: one similar to the 100-mg aspirin tablet and the other similar to 300-mg tablet. Patients and investigators could identify the size of the tablet without knowing the assignment arm (aspirin or placebo). Patients are randomized to one of the four treatment conditions: aspirin 100 mg, aspirin 300 mg, placebo of aspirin 100 mg, and placebo of aspirin 300 mg, according to a ratio 1: 1: 1: 1 using a previously established randomization standardized protocol. A total blind would have required the daily intake of 2 different tablets (1 active and 1 placebo or 2 placebos). This choice was made to favor compliance by reducing the number of tablets prescribed. Patients and investigators can therefore identify the size of the tablet but without knowing the assignment arm (aspirin or placebo) (protocol version 3.0 dated 2 June 2017).

Circumstances under which unblinding is permissible are severe digestive hemorrhage, traffic accident, and another situation requiring knowing if the patient is on aspirin. The procedure for revealing a participant’s allocated intervention during the trial consists in calling the project manager or, in case of emergency, the poison control center at Fernand Widal Hospital.

### Primary end-point

The primary end-point is defined as the number of patients with at least one adenoma detected during planned surveillance colonoscopies with chromo-endoscopy at 24 and 48 months. Additional colonoscopies will be performed as required after initial colon clearance at inclusion with complete polyp removal.

### Secondary end-points

The secondary end-points are defined as the adenomatous polyp burden (calculated as the sum of adenomatous polyps diameters as measured during planned surveillance colonoscopies); the delay between the inclusion and the first observed adenoma; the number of patients who presented with an adenoma during the follow-up according to the affected gene (MLH1, MSH2, MSH6, PMS2, other or no abnormality identified); the number of patients with sessile serrated adenoma, colon cancers diagnosed, and post-colonoscopy colon cancer (diagnosed between two planned surveillance colonoscopies); and time of apparition.

The quality of bowel cleansing, the number of chromo-colonoscopies performed in follow-up sessions, the number of remaining tablets in blisters, and side effects are reported.

### Participant timeline

#### Screening visit

The selection period takes place up to 180 days before the inclusion visit. The investigator collects the patient’s personal history: characteristics of LS, genetic test results, and colonoscopy results. During this screening visit, the informed consent is given with the information notice.

#### Visit V0

The investigator first will obtain written consent from all patients. Inclusion and exclusion criteria will be checked. The first colonoscopy will be performed no longer than 180 days before the inclusion visit to assess pre-existing lesions. Colonoscopies will be performed according to INCa (Institut National du Cancer) recommendations. Colonoscopies may be performed outside the investigator’s hospital, including any public or private institutions. All identified polyps will be removed during colonoscopies prior to the inclusion visit. During each colonoscopy, patients are scored against quality criteria as defined by the Digestive Endoscopic French Society (SFED) guidelines [19]: adequately prepared bowel (Boston ≥ 7), cecal intubation rate ≥ 90% (complete visualization of the cecum and appendicular tripod, endoscope terminal located in the proximity of the appendiceal orifice confirmed by a photo or a video), appropriate polypectomy technique (biopsy forceps removal for polyps ≤ 3 mm in size and snare polypectomy for larger polyps), and withdrawal time ≥ 6 min. Adenomatous, sessile serrated adenoma and polyp burden will also be collected.

#### Visit V1

During this visit (2 months after the randomization), stool will be collected using the OMNIgen®-GUT kit, blood drops deposited on blotting paper, data quality estimations made using the SF36 questionnaire, a survey of food ingestion frequency will be performed to describe dietary habits (SU-VI-MAX2 U557 Inserm) [20], a questionnaire investigating physical activity will be completed [21], and a urine pregnancy test realized. Patients will be randomly assigned a sample condition and scheduled to commence treatment the same day.

#### Follow-up visits

Six follow-up visits, interspersed at 6-month intervals, will be scheduled for the 4-year follow-up period: M6, M12, M18, M24, M30, and M36 (Table 2). During these visits, the patient will be provided with the next course of medication and compliance will be monitored. Medical examinations will be performed and additional colonoscopies scheduled as necessary. Urine pregnancy tests will be performed as described previously. Patients will be asked to report on any side-effects they had experienced as well as if they are undergoing other treatments, including self-medication. Further compliance and side-effect data will be collected, via telephone call, approximately 3 months before each follow-up visit.

**Table 2:** Research timeline

	V0, inclusion	V1, M0	V2, M6	V3, M12	V4, M18	V5, M24	V6, M30	V7, M36	V8, M42	V9, M48
Protocol information	**X**									
Informed consent	**X**									
Antecedent	**X**									
Clinical exam	**X**		**X**	**X**	**X**	**X**	**X**	**X**	**X**	**X**
Blood analysis	**X**									
Chromo-colonoscopy	**X (< 6 months)**			**If necessary**		**X**	**If necessary**			**X**
Stool collection		**X**								**X**
Nutritional survey	**X**	**X**								
Physical activity survey	**X**	**X**								
Quality of life survey		**X**								**X**
Blood drops blotting paper		**X**								
Pregnancy test		**X**	**X**	**X**	**X**	**X**	**X**	**X**	**X**	**X**
Randomization		**X**								
Treatments dispensation		**X**	**X**	**X**						
Adverse effects			**X**	**X**	**X**	**X**	**X**	**X**	**X**	**X**
Compliance observation			**X**	**X**	**X**	**X**	**X**	**X**	**X**	**X**

#### End-of-study visit

Approximately 48 months after inclusion, a final colonoscopy with chromoendoscopy will be performed, as in previous visits, medical examinations and blood analyses performed. Quality of life will be assessed on the SF36 questionnaire. An exclusion period will restrict patient enrollment in another trial for the 3-month period succeeding the trial. A summary table of the study timeline is available in Table 2.

### Biological collections

Biopsies will be performed for each patient. Tissue samples and smears will be prepared from samples taken during colonoscopies and then sent by post to the coordination centers for storage. Stool samples (prepared at home using an OMNIgen®-GUT kit) will be taken at the beginning and end of the treatment period. Samples will be labeled with an inclusion number and issue date then securely posted to a diagnostic/storage center. Blood samples will be collected on a blotting paper from which DNA will be subsequently extracted.

### Recruitment

Patients will be recruited from 34 French specialist institutes with dedicated oncogenetic teams managing Lynch patients. Almost 3000 patients are registered with these centers. All patients are tested for gene alterations and MMR deletions and are monitored closely. The HNPCC-Lynch Patient Association will assist in the recruitment of patients to this study by notifying its subscribers about the study’s existence.

### Statistical methods

The initial characteristics of the population will be described globally and by group (age, sex, BMI, MSI–MLH1; MSH2; MSH6; PMS2–). The continuous variables will be described using average, standard deviation, median, minimum, and maximum. The qualitative variables will be described using frequency and percentages.

The main analysis will be done in intention of treat. A per-protocol analysis will also be performed. The statistical significance level will be 5% and all tests will be bilateral.

The main end-point analysis will be performed using logistic regression with age (< and ≥ 45 years) and using center adjustment. A specific analysis of the dose effect will be performed between the two arms of aspirin (100 mg vs 300 mg) in the case of an overall effect in the aspirin group. A subgroup analysis will be performed according to age and the *p* value was corrected according to the Bonferroni method. A center effect analysis effect will also be performed when institutes provided a small number of patients. An intermediate analysis will be carried out after 24 months of treatment on secondary judgment criteria. The Kaplan-Meier method and a log-rank test will be used to assess the effect of treatment on the delay of the appearance of the first adenoma. Patients who presented an adenoma during follow-up according to MMR status, colon cancers diagnosed, and post-colonoscopy colon cancers will be evaluated by logistic regression with age and center adjustment. Sessile serrated adenoma and adenomatous burden will be evaluated using a Poisson or a binomial regression approach.

All of the patients included in the study will be considered in the analysis. For the main analysis, lost subjects will be considered failures, regardless of the treatment group. Sensitivity analyses will also be performed, using multiple imputation methods.

### Sample size

The main objective of this trial is to show that adenoma prevalence is less important in the aspirin group (regardless of the dose) than in the placebo group. The initial hypothesis was constructed with a 30% adenoma prevalence in the placebo group and a reduction of 28% expected in the aspirin group, based on our previous RCT on patients without Lynch syndrome but with a history of colorectal adenomas [8]. For a 5% alpha risk and 80% power, according to this hypothesis, 426 patients will be needed in the aspirin group (213 patients for aspirin 100 mg arm and 213 patients for aspirin 300 mg arm) and 426 patients in the placebo group (213 patients for placebo aspirin 100 mg arm and 213 patients for placebo aspirin arm 300 mg). A total of 852 patients will be included.

## Discussion

Aspirin inhibits the two cyclooxygenase enzyme isoforms COX-1 and COX-2 which catalyze the rate-limiting step in the metabolic conversion of arachidonic acid to prostaglandins and related eicosanoids, leading to an inhibition of prostaglandin synthesis. COX-1 is constitutively expressed in the colon whereas COX-2 is inducible and promotes inflammation, cell proliferation, and survival. The putative anticarcinogenic effect of aspirin is therefore thought to be mainly linked to COX-2 inhibition. Aspirin irreversibly inactivates platelet cyclooxygenase in the systemic circulation, which translates into a long-lasting inhibition of platelet function. A recent study showed the role of activated platelets in colorectal tumorigenesis and dissemination through direct cell-cell interactions, lipid and protein mediators release which could be an additional target to aspirin [22]. Aspirin inhibits proliferation and induces apoptosis of cancer cells in vitro, independently of its inhibitory effect on prostanoid biosynthesis [23].

There is considerable evidence that aspirin can reduce the risk of colorectal cancer in the general population [4, 6, 8, 10–12]. However, some studies, including recently conducted randomized trials, did not characterize any protective effect of aspirin on colorectal cancer in a specific population [9, 13, 14]. Aspirin use over 4.7 years in a population of elderly, but healthy individuals, yielded an unexpected increase in the number of colorectal cancer-related deaths in contrast with previous RCT [24]. Various hypotheses have been formulated to explain these discrepancies, in particular, that biological aspirin effects may vary according to the timing of exposure, especially if undiagnosed tumors are already present which is often the case in elderly patients.

In LS patients, the randomized CAPP2 trial did not show any aspirin protective effect on colorectal adenoma or cancer incidence after a mean of 29 months, raising the suspicion that the distinct mismatched neoplastic pathway could be less susceptible to protection by aspirin [15]. However, when the follow-up reached a mean of 56 months, a significant reduction in cancer incidence was observed (1 in the aspirin group and 4 in the placebo group; IRR 0.56, 95% CI [0.32–0.99] *p* = 0.05) [16]. Confirmation of these results would strengthen aspirin’s chemopreventive potential in LS patients even if wide-spread aspirin usage is encouraged. An ongoing CAPP3 trial is exploring the optimal dose of aspirin by randomizing 3000 LS patients to 100, 300, and 600 mg/day but without considering a placebo group. The AAS-Lynch trial could contribute to these evaluations and also determine the most effective dose between 100 and 300 mg conditions. Aspirin’s chemopreventive effect could contribute to the reduction in the burden of endoscopic surveillance in Lynch patients according to different germline alterations in mismatch repair genes. The AAS-Lynch trial will report high-quality colonoscopy details and will allow delineating such a benefit. Long-term aspirin safety is already known [25] but has been reevaluated in this specific population [25]. Furthermore, this study will also allow describing compliance with respect to chemoprevention by daily aspirin therapy in Lynch syndrome patients.

The gut microbiota is a complex community consisting of bacteria, fungi, protozoa, viruses, and bacteriophages which live in a symbiotic and epigenetic relationship with the host. Gut microbiota can promote either health or tumor progression through its inflammatory and proliferative effects, depending on the context and genetic factors of the host. Specific microorganisms or variability of the microbiota have been recently associated with CRC but how gut microbiota contributes to CRC pathogenesis in the host is not fully understood. This prospective study design would account for the nutritional habits of the population, include validated questionnaires, and collect stool samples in order to investigate to the role of microbiota in colorectal carcinogenesis.

### Trial status

AAS-Lynch protocol version number is 3-0, from June 2, 2017. The date of the first enrolment is November 14, 2017. The estimated date of primary completion is December 2024. Detailed trial registration data set is presented on Table 3.

**Table 3:** Trial registration data set

Data category	Information
Primary registry and trial identifying number	ClinicalTrials.gov NCT02813824
Date of registration in primary registry	June 27, 2016
Other study ID numbers	P130937
Source(s) of monetary or material support	Programme Hospitalier de Recherche Clinique National (P130937) La Fondation ARC Bayer Laboratory
Primary sponsor	Programme Hospitalier de Recherche Clinique National (P130937)
Secondary sponsor(s)	La Fondation ARC Bayer Laboratory (investigational product)
Contact for public queries	Amal BOURKEB amal.bourkeb@aphp.fr
Contact for scientific queries	Pr Robert BENAMOUZIG robert.benamouzig@aphp.fr
Official title	Assessment of the Effect of a Daily Chemoprevention by Low-dose Aspirin of New or Recurrent Colorectal Adenomas in Patients With Lynch Syndrome
Countries of recruitment	France
Health condition(s) or problem(s) studied	Lynch syndrome
Intervention(s)	Aspirin low dose (100 or 300 mg) versus placebo in lynch syndrome patients
Key inclusion and exclusion criteria	Ages eligible for study: ≥ 18 years Sexes eligible for study: both Accepts healthy volunteers: no Inclusion criteria: Lynch syndrome patients aged over 25 or 18 years of age in cases with an early family history, with an indication for colonoscopic surveillance every 2 years, under 75 years old, performing a colonoscopy within 180 days before inclusion, with removal of all endoscopically resectable polyps, no regularly use of aspirin… Exclusion criteria: Total colectomy, adenomatous polyposis associated with known alteration of APC gene or MYH gene, allergy to aspirin, allergy known to indigo carmine used for chromoendoscopy, indication of prolonged treatment or repeated treatment with aspirin or other nonsteroidal anti-inflammatory drug, abnormality of hemostasis or coagulation…
Study type	Interventional Allocation: randomized Parallel assignment Masking: Quadruple (participant, care provider, investigator, outcomes assessor) Primary purpose: Prevention Phase III
Date of first enrolment	November 14, 2017
Estimated primary completion date	December 2024
Estimated study completion date	December 2025
Protocol version number and date	Version 3-0; June 2, 2017
Target sample size	852 participants
Recruitment status	Recruiting
Primary outcome(s)	Number of patients with at least one adenoma seen on chromo-endoscopy 48 months after complete withdrawal of polyps and initiation of treatment (aspirin or placebo) [time frame: 4 years]
Key secondary outcomes	Delay between the onset of 1 adenoma after complete resection of polyps and date of start of treatment (aspirin vs placebo) Number of patients who presented an adenoma during follow-up based on the gene reached (MLH1, MSH2, MSH6, PMS2, or without other identified anomalies) Load serrated polyps after 24 and 48 months of treatment

### Steering and scientific committee

The steering committee is composed by the coordinating investigator, at least one other investigator, the biostatistician, and the project manager. It meets twice a month. Its role is to design the protocol, the manual of procedures, and write all the documents of the study (information notice, consent form, newsletter, etc.). It ensures the study progress, makes decisions relating to the choice of new investigators and means to be implemented to ensure the recruitment. It follows the inclusions’ progression and suggests solutions if there are recruitment difficulties. The committee reviews the execution of these decisions throughout the trial through clinical research associates.

The scientific committee meets twice a year and is responsible of the trial direction, from conception to publication. It is involved in the conduct and translational research. Any modification to the protocol needs its approval. It is responsible for writing and possibly publishing the protocol before the end of the trial. The committee is composed of the principal investigator, some clinicians practicing in the specialty concerned by the trial, pathophysiology searchers, pharmacologists, biostatisticians, methodologists, statisticians, data managers, and a representative of the coordination center. The committee answers all questions regarding ethical, medical, and scientific issues, to provide clinical research associate with any new information concerning the study.

## Data Availability

The datasets used and analyzed during the current study are available from the principal investigator (corresponding author) on reasonable request.

## References

[1] Vasen HFA (2010). One to 2-year surveillance intervals reduce risk of colorectal cancer in families with lynch syndrome. Gastroenterology.

[2] Chan AT, Ogino S, Fuchs CS (2007). Aspirin and the risk of colorectal cancer in relation to the expression of COX-2. N Engl J Med.

[3] Rothwell PM (2010). Long-term effect of aspirin on colorectal cancer incidence and mortality: 20-year follow-up of five randomised trials. Lancet.

[4] Baron JA (2003). A randomized trial of aspirin to prevent colorectal adenomas. N Engl J Med.

[5] Sandler RS (2003). A randomized trial of aspirin to prevent colorectal adenomas in patients with previous colorectal cancer. N Engl J Med.

[6] Logan RFA (2008). Aspirin and folic acid for the prevention of recurrent colorectal adenomas. Gastroenterology.

[7] Pommergaard H-C, Burcharth J, Rosenberg J, Raskov H (2016). Aspirin, calcitriol, and calcium do not prevent adenoma recurrence in a randomized controlled trial. Gastroenterology.

[8] Benamouzig R (2003). Daily soluble aspirin and prevention of colorectal adenoma recurrence: one-year results of the APACC trial. Gastroenterology.

[9] Hull MA, Sprange K, Hepburn T (2018). Eicosapentaenoic acid and aspirin, alone and in combination, for the prevention of colorectal adenomas (seAFOod Polyp Prevention trial): a multicentre, randomised, double-blind, placebo-controlled, 2 × 2 factorial trial. Lancet.

[10] Ishikawa H (2014). The preventive effects of low-dose enteric-coated aspirin tablets on the development of colorectal tumours in Asian patients: a randomised trial. Gut.

[11] Cole BF (2009). Aspirin for the chemoprevention of colorectal adenomas: meta-analysis of the randomized trials. JNCI J Natl Cancer Inst.

[12] Sutcliffe P (2013). Aspirin for prophylactic use in the primary prevention of cardiovascular disease and cancer: a systematic review and overview of reviews. Health Technol Assess Winch Engl.

[13] The ASCEND Study Collaborative Group (2018). Effects of aspirin for primary prevention in persons with diabetes mellitus. N Engl J Med.

[14] McNeil JJ (2018). Effect of aspirin on all-cause mortality in the healthy elderly. N Engl J Med.

[15] Burn J, Bishop DT, Mecklin JP, et al. Effect of aspirin or resistant starch on colorectal neoplasia in the Lynch syndrome [published correction appears in N Engl J Med. 2009 Apr 2;360(14):1470]. N Engl J Med. 2008;359(24):2567–78. 10.1056/NEJMoa0801297. 10.1056/NEJMoa0801297 19073976

[16] Burn J (2011). Long-term effect of aspirin on cancer risk in carriers of hereditary colorectal cancer: an analysis from the CAPP2 randomised controlled trial. Lancet Lond Engl.

[17] Järvinen HJ (2000). Controlled 15-year trial on screening for colorectal cancer in families with hereditary nonpolyposis colorectal cancer. Gastroenterology.

[18] Rahmi G (2015). Impact of chromoscopy on adenoma detection in patients with Lynch syndrome: a prospective, multicenter, blinded, tandem colonoscopy study. Am J Gastroenterol.

[19] Bernardini D, Lapuelle J, Chaussade S, Robaszkiewicz M (2019). Critères de qualité du compte rendu de coloscopie – Recommandations du CNP-HGE et de la SFED. Hépato-Gastro Oncol Digest.

[20] Hercberg S (2004). The SU.VI.MAX study: a randomized, placebo-controlled trial of the health effects of antioxidant vitamins and minerals. Arch Intern Med.

[21] Golubic R (2014). Validity of electronically administered recent physical activity questionnaire (RPAQ) in ten European countries. PLoS One.

[22] Patrignani P, Patrono C (2018). Aspirin, platelet inhibition and cancer prevention. Platelets.

[23] Dovizio M, Bruno A, Tacconelli S, Patrignani P (2013). Mode of action of aspirin as a chemopreventive agent. Recent Results Cancer Res.

[24] Chan AT, McNeil J (2019). Aspirin and cancer prevention in the elderly: where do we go from here?. Gastroenterology.

[25] Fanaroff AC, Roe MT (2016). Contemporary reflections on the safety of long-term aspirin treatment for the secondary prevention of cardiovascular disease. Drug Saf.

